# The practicality of implementing a net energy system in the broiler industry

**DOI:** 10.1016/j.aninu.2026.07.001

**Published:** 2026-10-09

**Authors:** Mingan Choct, Shubiao Wu

**Affiliations:** School of Environmental and Rural Science, University of New England, Armidale 2351, NEW, Australia

**Keywords:** Broiler chicken, Metabolisable energy, Net energy, Nitrogen-correction

## Abstract

There is momentum for the poultry industry to transition from an apparent metabolisable energy (AME) system to a net energy (NE) system for diet formulation. This review is not a rehash of the in-depth details of the feed energy system. Rather, it focuses on the practical issues related to the broiler chicken industry's transition to NE, such as the various terminologies associated with metabolisable energy (ME) and their implications, heat increment (HI) and factors that affect it, the use of NE and the impact of feed additives, and the correct use of the key NE equations commonly recommended for poultry.

## 1 Introduction

Energy is the most expensive part of a poultry diet, and it is the parameter on which all other nutrients are anchored during feed formulation. Yet energy is perhaps the item on a nutritionist's database that carries the most uncertainty. The uncertainty arises primarily from the fact that energy is not the sole characteristic of feed; it is not a nutrient, nor is it contained in every nutrient. It is an item resulting from the interaction between the energy-bearing components of feed and the animal that consumes it. This makes the determination of the energy value of feed, in particular, that of individual ingredients that make up a formulated diet, highly complicated. The reasons are multifaceted, including the species of the animal and its unique digestive system, the intrinsic chemical and physical characteristics of the feed, the age and physiological state of the animal, and the method by which the energy value is obtained.

The process of energy utilisation in poultry can be broken down into two phases based on how the industry sets the energy value of feed. The first phase is from gross energy (GE) to metabolisable energy (ME). Metabolisable energy is a punctuation point for feed energy in poultry as it is used as the default system for the energy value of feed in poultry feed formulation. The second phase is from ME to net energy (NE). Net energy is the final stage of feed energy journey in animals, where retained energy and maintenance energy can be partitioned and quantified. This partitioning is useful for increasing the precision of matching nutrient requirements with the amount of energy available for production (muscle growth, fat accumulation, or egg output). Indeed, there is strong momentum for the poultry industry in many parts of the world to transition from an ME to an NE-based formulation, especially for broiler chickens. However, several technical and logistical hurdles exist in implementing an NE system due to conflicting information in the literature as to whether NE values of individual ingredients should be tabulated or simply calculated using prediction equations, and the question of whether the NE system is beneficial compared to ME. If equations are to be used, of the impact of feed additives, such as enzymes, on the accuracy of prediction, in particular when it comes to the use of high levels of fibre- and protein-rich ingredients.

This review focuses on some practical considerations regarding the use of the NE equations developed by Wu et al. (2019), Tay-Zar et al. (2024), and Noblet et al. (2022, 2024) for feed formulation for broiler chickens. The importance of an accurate ME value and the impact of nitrogen correction will be outlined, and the role of fibre discussed. Furthermore, it will clarify the confusion over some of the technical details of the equations, ie., dry matter (DM) vs. as is, MJ vs. kcal, and recommend a broad guide for setting NE requirements. Finally, the benefit of the NE vs. apparent metabolisable energy (AME) system will be briefly discussed.

## 2 From GE to ME

In poultry, the AME is the current default system for dietary energy value. The AME refers to the difference between the gross (total) energy (GE) content of a feed and the energy content of the excreta (the mixture of faeces and urine). A very small amount of gaseous losses can occur in poultry, which is ignored. Thus, the AME is GE of feed minus GE of excreta. It is also the sum of retained energy (RE; protein and lipid, plus a negligible amount of carbohydrate in the form of glycogen) and heat production (heat increment [HI] and fasting heat production [FHP]), ie.,

AME=RE+HI+FHP.

As an example, one kg of poultry feed on a DM basis contains approximately 4600 kcal of GE, which yields 3450 kcal AME, with 1150 kcal energy lost in the excreta. This means that the efficiency of GE for ME is 75%, leaving 25% of a nutritionally balanced poultry diet lost in the excreta. The 25% loss of dietary energy represents energy excreted in the faeces (c. 1050 kcal) and in the urine (c. 100 kcal). The faecal energy loss varies markedly because ingredient digestibility differs, affecting the amount of undigested nutrients remaining in the faeces. Likewise, the urinary energy loss is influenced by the dietary protein level as it affects the amount of nitrogen (N) metabolised and excreted as uric acid. Wu et al. (2019) presented a set of 19 broiler diets that varied in ingredient composition and nutrient levels but all the diets supported breed-standard bird performance. In this study, the GE utilisation for AME averaged 73%, ranging from 66% to 80%.

In commercial feed formulation, 50 kcal is material (Xie et al., 2024). Public domain and proprietary feed tables present a mixture of AME and true ME (TME) but also the same concepts with adjustments for a zero N balance (AMEn and TMEn, respectively) (Noblet et al., 2022; Wu et al., 2020). The ME value of a single raw material can then be quite different, depending on where (origin of the data) the figure came from, how (broilers or adult birds) the data were obtained, and what (N corrected or not) form the value was presented (Mateos et al., 2019). For instance, the AMEn value of feeds is higher in adult cockerels than in fast-growing broilers (Cozannet et al., 2010), indicating that energy values obtained in adult birds, which are still widely used in feed tables, are not applicable to practical conditions with fast-growing broilers. For the broiler industry, this also means that AME measurements should be conducted in broilers in the mid-production cycle (Noblet et al., 2022).

Obtaining an accurate AME value for each batch of feed before large-scale manufacturing for commercial poultry feeding is theoretically possible but practically difficult. It is also of little technical and economic advantage to do so since the AME value of a feed is affected by the age of the animal (Zelenka, 1968), feed intake (Farrell et al., 1991; Sibbald, 1975; Zelenka, 1997), the growing environment of the ingredients (Choct et al., 1999), and the method of determination (Wu et al., 2020). In particular, the age at which the AME assay is conducted affects the AME values greatly, with older birds yielding a higher AME value than younger birds, especially for protein ingredients (Adeola et al., 2018; Jiang et al., 2024). In practice, however, measuring the ME content of a complete diet has limited value, except for cross-checking the accuracy of the formulation against the actual value. What is required is the ME values of individual ingredients that make up a compound feed, but assaying the ME values of individual ingredients is complex. Firstly, the test diet needs to meet the basic requirements of the animal, although a perfectly balanced commercial standard feed is often too much to ask for in an ME assay diet. The lower the inclusion level of the test ingredient, the closer to a balanced diet, but the higher the margin of error of determination. Thus, in order of difficulty, the level of difficulty for an AME assay starts with fats and lipids, which come first being most difficult, followed by proteins, and then cereal grains. As cereal grains are included in high levels to meet the energy requirements of poultry, the margin of error is usually low, regardless of the assay method used. However, ingredients such as vegetable protein meals, animal by-products, and fats and oils cannot be included in large quantities in formulations without disturbing the nutrient balance of the diet. In addition, the impact of age on the utilisation of fat sources is significant (Wiseman et al., 1998). It is due to young bird's immature digestive system, which is unable to secrete a sufficient amount of bile acids to effectively emulsify saturated fat and produce enough lipase to degrade fat to fatty acids (Wiseman, 1990). Other factors, such as the ratio of saturated to unsaturated fats, level of oxidation during storage and processing, free fatty acid content, unknown blends of fats and oils of different origins (Wiseman and Salvador, 1991) and the degree of saturation, all affect the ME content of fats and oils for poultry (Krogdahl, 1985; Ravindran et al., 2016). Indeed, the choice of assay method matters a great deal. Inappropriate bioassays, unnecessary adjustments, poor experimental designs, and flawed statistical or mathematical calculations are the main reasons for the large variation that exists in ME values in the literature (Wu et al., 2020). Some of the key factors that influence the AME value of feed ingredients are comprehensively covered in Wu et al. (2020) and Noblet et al. (2022).

In industry settings, the determination of the AME value of all new ingredients is often not realistic. Thus, the most practical way to improve the accuracy of AME values for commercial feed formulation is to carefully check the origins of the major raw materials to determine whether they were N-corrected, obtained from adult or growing birds, and assayed as AME or TME. Of course, if conditions permit, ongoing cross-checking of the formulated vs. determined AME values, as well as the measurement of the raw material AME values using an appropriate bioassay, will be the ultimate quality assurance (QA) work for dietary energy. For a limited number of ingredients, near-infrared (NIR) methods allow a direct estimation of AMEn values. Unfortunately, NIR calibration datasets are often proprietary and hence not available as public domain information. Noblet et al. (2022) reported a few equations based on chemical composition that could be used to predict the GE, AME, and AMEn values of feed ingredients. But the accuracy of the prediction is highly dependent on the availability and the accuracy of the chemical indicators and it does not capture the ingredient-specific effects on digestibility.

The most common source of energy values is the feed tables (CVB, 2026 [https://images.engormix.com/externalFiles/6_cvb-feed-table-2016-version-1.pdf]; INRAE-CIRAD-AFZ, 2026 [https://feedtables.com]; Brazilian tables 2024 [https://appec-h.com/wp-content/uploads/2024/08/Brazilian-Tables-for-Poultry-and-Swine-4th-ed.pdf]; or FEDNA, 2021 [https://www.fundacionfedna.org/tablas-fedna-composicion-alimentos-valor-nutritivo]). Popular feed tables present ingredient energy values as AMEn. Correcting AME for zero N retention is not relevant in growing broilers as they retain 40% to 70% of their N intake under field conditions (Noblet et al., 2022). However, the reality is that most poultry nutritionists rely on book values and hence use AMEn, rather than AME values in feed formulation.

## 3 From ME to NE

The AME is the sum of energy retained (RE) as protein and fat (a small amount of carbohydrate ignored), HP for maintenance and HP for feeding (also known as HI). In practice, HP for maintenance is determined as fasting HP (FHP), ie., when the animal is not consuming feed, is kept at thermoneutrality and with no physical activity. Armsby and Fries (1915) defined NE as the difference between ME and HI. They thought that HI was independent of level of feeding and thus it could be tabulated. It is now understood that HI is affected by feed intake and is not the sole characteristic of feed. Nor can it be easily predicted based on nutrient composition, although there are well-accepted methods (the comparative slaughter method and indirect calorimetry) for estimating NE values of feed. Noblet et al. (2022, 2024) discussed the NE system(s) for poultry in detail, where they presented that the efficiency of converting AME to NE averages around 75%–76% in growing broilers. This means that HI represents approximately a 25% loss of ME as heat during the digestion, absorption, and metabolism of nutrients, and at a standard level of activity. The magnitude of energy loss (c. 25%) as heat in converting AME to NE is similar to that in converting GE to AME. The comparison, however, is not as straightforward as it may look in terms of the potential to minimise the loss through manipulation of feed formulation. The efficiency of GE for AME varies depending on the raw material in question and everything else that affects its digestion and subsequent absorption of the released nutrients. The extent of variation in AME values relative to GE of feed is large due mainly to the very small contribution that fibre makes to AME. Therefore, any improvement in the digestibility of the feed narrows the 25% gap between GE and, for example, by reducing fibre content or increasing the digestibility of the other energy-bearing nutrients. The energy loss from AME to NE, ie., HI, on the other hand, is determined by the feed or ingredient content of crude protein (CP), ether extract (EE; crude fat), and even fibre content as shown in recent studies (Noblet et al., 2024; Wu et al., 2019; Tay-Zar et al., 2024). Although there is potential to manipulate the levels of these nutrients in formulation, such as reducing the protein level, increasing the fat content and modulating the fibre usage through the use of enzymes, it is challenging under normal commercial settings. Furthermore, the coefficients of these nutrients in the NE equations are small. Also, the variation of NE values relative to AME values is not as large as that of AME relative to GE. Therefore, there is a perception among some researchers and industry nutritionists that moving from AME to NE may not be worthwhile due to their familiarity with, and the availability of data for, AME, coupled with the apparent lack of obvious benefits of using NE.

The energy journey from AME to NE appears simple, but in reality, it is highly complex. The variation in heat production during the utilisation of AME, for instance, arises from numerous fundamental life processes. In chickens, it starts from the moment the bird stands up and pecks at the feed, followed by swallowing, salivary secretion, digestion, accretion of body tissues (protein and fat), or producing eggs, to excretion of faeces and urine (Emmans, 1994). The 25% HI loss excludes the energy expenditure associated with maintenance, i.e., when the animal is at zero intake. The current NE systems measure FHP to determine the maintenance heat production. In the studies resulting in the development of the most recent equations (Wu et al., 2019; Noblet et al., 2024; Tay-Zar et al., 2024), a single value of 450 kJ (108 kcal)/(kg BW^0.70^ ·d) for modern broiler chickens such as Ross 308 was used, which was based on Noblet et al. (2015), who conducted six experiments (*n* = 73 in 3- to 6-week-old broilers) to obtain the FHP values at zero intake and activity with two different breeds. The FHP values from the six experiments ranged from 410 to 489 kJ/(kg BW^0.70^ ·d). A distinct feature of modern industrialised broiler production systems is that a set of husbandry and management conditions is recommended. Despite the fact that adhering to such recommendations is not always possible due to numerous factors, a level of uniformity in rearing conditions for broiler chicken production across different countries and regions is expected. Thus, it is not unreasonable for Wu et al. (2019) and Noblet et al. (2024) to adopt the approach taken to use a single FHP value for the development of the NE equations recommended in this review.

From a broad class nutrition point of view, the impact of dietary protein and fat on HI in broiler chickens is well characterised (Wu et al., 2019; Tay-Zar et al., 2024). It is also certain that HI is not a constant for the same feed, but the extent to which HI changes due to bird and environmental factors in the modern broiler chicken would not alter the ranking of ingredients. Heat increment is not uniform across raw materials, nutrients, or indeed individual birds. However, as a general rule, heat production is high for fibrous raw materials and vegetable protein sources, intermediate for starchy ingredients, and low for fats and oils (Noblet et al., 2024; Wu et al., 2019; Tay-Zar et al., 2024). It is highly tedious, costly and impractical to determine HI for every raw material that becomes available in a feed mill. This brings about the necessity to use prediction equations for NE. Albeit the limitations of this approach, namely, the inability to offer NE values specific to certain age brackets, production conditions or breed, an NE system offers an improvement on AME-based systems as it takes into account additional variation associated with HI. Net energy supply from feed more accurately reflects the energy requirements of birds than AME, thereby minimising potential imbalances between amino acids and energy that may arise when the AME requirement of birds is used, particularly when diets differ in protein and fat contents.

## 4 Common questions related to the use of NE equations

As energy costs escalate, many producers have begun exploring the use of an NE system for feed formulation. However, several key hurdles, both technical and practical, remain regarding the use of the equations (Noblet et al., 2022, 2024; Wu et al., 2019; Tay-Zar et al., 2024).

1) AME vs. AMEn

Wu et al. (2019) recommend key equations for estimating NE values of broiler diets. The two equations, one based on AMEn (Eq. [1]) and another based on AME (Eq. [2]) are:

(1) NE(MJ/kgDM)=0.808×AMEn(MJ/kgDM)–0.017×CP(%)+0.031×EE(%)

(2) NE(MJ/kgDM)=0.781×AME(MJ/kgDM)–0.028×CP(%)+0.029×EE(%)

To the untrained person, the various terminologies used to describe the ME values of feed can be quite confusing. Despite controversies surrounding the correction of AME values to zero N balance, most feed tables give AMEn values (CVB, 2026; INRAE-CIRAD-AFZ, 2026; Rostagno and Albino, 2024). Thus, Eq. (1) is most relevant for the majority of broiler chicken companies using table values. There are companies that have their own facility or have access to such a facility to measure the AME and AMEn values of all the ingredients in use. This enables them to use either of the Wu et al. (2019) equations presented above in their transition to an NE-based formulation system.

2) DM vs. as is, units of the equations

Industry users are often unsure about how the units in the equations should be used. For instance, questions often arise whether all the database energy values should be converted to dry matter basis first, and whether the % CP and EE values should be converted. Of course, all the published equations used values based on dry matter to reduce errors and ensure consistency. But under normal industry conditions, as the moisture levels of ingredients stay within a reasonable range, the equations remain valid on as is basis without having to convert the data to DM basis. Likewise, the % sign in brackets for the CP and EE values simply indicates that the coefficients are for percent CP and EE (crude fat) values and hence they are not to be adjusted.

3) MJ vs. kcal

From the 1970s, some countries started to adopt MJ as the standard unit for feed energy, driven by the shift towards the SI units in scientific literature and international feeding standards. However, the transition from kcal to MJ has not been uniform across the globe, with most countries still using kcal. The relationship between the two units is straightforward:

1MJ=239.00kcal.

1000kcal=4.184MJ.

For countries and regions that use kcal, the Wu et al. (2019) equations (presented as Eq. (3) and Eq. (4) earlier) are given below in kcal/kg:

(3) NE(kcal/kg)=0.808×AMEn(kcal/kg)–4.06×CP(%)+7.41×EE(%)

(4) NE(kcal/kg)=0.781×AME(kcal/kg)–6.69×CP(%)+6.93×EE(%)

4) Effects of feed additives

Whether the NE equations have taken into account the impact of feed additives such as enzymes is a vexed question. It is often difficult to decipher when the impact of feed additives on ME ends and that on NE commences. On the surface, the effect of feed additives on ME should be captured in increases in nutrient digestibility, even though ME goes beyond digestion and actually includes urinary energy output, which concerns the absorption of nutrients. However, HI associated with digestion and absorption cannot be completely partitioned because life processes that produce heat do not have a demarcation between AME and NE. In fact, feed additives that enhance gut health, modulate immunity and remove anti-nutrients will have benefits that cannot fully be materialised solely in apparent digestibility increases. However, from a practical point of view, partitioning the energy matrix values for additives, such as enzymes, into those brought about by ME and NE is futile at this stage of development.

Wu et al. (2019) used phytase and xylanase in all 19 diets for their prediction equation development. Tay-Zar et al. (2024) also used phytase and xylanase in the 23 diets that they used to develop their NE prediction equations. Noblet et al. (2024) presents a compilation of published literature on NE in poultry, covering a range of diets with or without enzymes. Xylanase is one of many carbohydrases used in poultry feed and therefore, the impact of many other carbohydrases is yet to be determined. Logically speaking, inducing structural changes to non-starch polysaccharides (NSP) by the use of enzymes will do one or both of the following: reduce the negative effects of NSP on nutrient digestion and absorption (Choct and Annison, 1992) or produce prebiotics in situ to enhance the gut health of the animal (Morgan et al., 2019). Another class of enzymes increasingly used in poultry feed is proteases. The main purpose of using protease is to increase the digestibility of proteins that are either poorly digested or not digested at all by the endogenous proteases present in chickens (Toghyani et al., 2017). This would help reduce heat production and increase the NE value of feed. In addition, it is postulated that other benefits could arise from the use of protease, such as the degradation of antigenic proteins, alleviating stress on the gut and the immune system. Indeed, Cowieson et al. (2019) demonstrated a larger magnitude of improvement in NE (+108 kcal) than in AME (+72 kcal) in broiler diets with supplemental protease. This shows that protease supplementation has a proportionally greater impact on NE. McCafferty et al. (2022) further confirmed it by showing that protease supplementation reduced HI from 431 to 394 kJ/kg BW^0.70^ in broiler chickens fed a conventional diet, suggesting that protease supplementation had an effect on NE.

To the best of our knowledge, none of the equations published in recent times took into account the impact of other additives such as acids, phytobiotics, probiotics, and prebiotics, etc., but the impact of these additives on NE is outside the scope of the current review.

5) Impact of fibre

Fibre in animal nutrition has been a confusing moiety because the prevailing view of “fibre” has been crude fibre (CF), which doesn't represent the true fibre content of feed ingredients. Further confusion about fibre has arisen because of the tremendous global effort to remedy the inadequacy of CF in animal nutrition by coming up with various fibre entities, such as neutral detergent fibre (NDF), acid detergent fibre (ADF), structural fibre and functional fibre etc. From an animal nutrition point of view, the sum of NSP and lignin, defined as total dietary fibre (TDF), adequately covers the true fibre content of feed. Noblet et al. (2024) proposed an equation that contained NDF as a factor:

NE(MJ/kgDM)=0.815×AME–0.026×CP+0.020×EE–0.024×NDF;

or

NE=(kcal/kgDM)=0.815×AME(kcal/kg)–6.21×CP+4.78×EE–5.74×NDF.

Although NDF values for cereals and cereal by-products are similar to the TDF contents, those for ingredients containing high levels of pectic polysaccharides, i.e., vegetable protein sources, are not so (Choct, 2015). Noblet et al. (2024) did not have access to a readily available TDF database to use. However, the recognition of the roles of fibre in NE is perhaps important. In a properly formulated broiler diet, for instance, the CF level ranges between 30 and 40 kg/t, whereas NDF or TDF (non-starch polysaccharides + lignin) content ranges between 100 and 140 kg/t.

Fibre is often regarded as a double-edged sword in poultry nutrition because some soluble fibre moieties can be antinutritive (Choct et al., 1992), whereas fibre with particular physical consistencies (coarse, durable) can enhance gizzard functions and gut health (Hetland et al., 2019). Furthermore, fibre is by far the most important source of prebiotics (Bautil et al., 2020; Choct et al., 2004) and appropriate supplemental enzymes can lead to the production of prebiotics in situ, which improve gut health and enhance bird performance (Morgan, 2023). The gut is the largest immune organ in the body of the chicken and the immune system accounts for about 9% of nutrient needs under non-challenged conditions (Klasing, 2007). It is difficult to translate this figure into an HI value. For argument's sake, for a broiler grower diet that contained 3020 kcal ME/kg, the immune system would require 270 kcal. A large proportion of the energy cost appears to be related to maintaining the basic metabolic rate, an important part of heat production. Martin et al. (2003) measured the metabolic cost of an immune challenge in house sparrows (*Passer domesticus*). They found that activation of the immune system (using phytohaemagglutinin) increased resting metabolic rate by about 29% relative to baseline, indicating immune activation costs in the order of 25%–30% of basal metabolic rate in that species of bird. It is unknown whether this figure can be extrapolated to the nutritional immunity of broiler chickens. Indeed, direct evidence suggests that carbohydrase supplementation, especially in broiler diets containing high levels of vegetable proteins, improves NE retention (Toghyani et al., 2017).

6) Reduced protein diets

Environmental, economic, and welfare concerns have prompted the monogastric animal industries to reduce the protein level in feed formulation. It appears that a reduction of up to 2 percentage points in dietary crude protein for broiler chicken diets has little or no effect on growth rate, FCR, and carcass fat content (Chrystal et al., 2020; Musigwa et al., 2024, 2025). According to industry experience, formulating on NE values leads to broiler diets having lower AME (40–80 kcal) and protein (0.5-1.5 percentage points) naturally (Srichana et al., 2025). This occurs only when dietary energy is set to NE without reference to ME, with amino acid levels set according to the ideal protein profile, balanced with synthetic amino acids, with proper consideration of the balance of both essential and non-essential amino acids as per breed recommendations. The number and types of ingredients available for formulation often determine how much reduction in dietary protein content is practically possible and economically feasible. In general, reducing dietary protein level increases metabolic and energetic efficiency, ie., reducing HI, but the actual energy savings resulting from a 1 to 2 percentage point reduction in CP content of a broiler diet are negligible. The potential benefits associated with the use of reduced protein diets will be further covered later.

7) NE requirements

The key feed tables give the AMEn values of ingredients and the energy values set in breed nutrient recommendations are given in AMEn. Therefore, the NE requirements for different phases of broiler growth can be derived from the recommended energy and CP levels, along with estimated fat levels, for the standard starter, grower, and finisher diets specified in the breed nutrition specifications. For example, Aviagen (2022) recommends the following ME and CP levels of starter, grower, finisher 1, and finisher 2 phases for as-hatched Ross broilers, targeting marketing live weights between 2.0 and 3.5 kg: ME, 2975, 3050, 3100, and 3125 kcal/kg; and CP, 23.0%, 21.5%, 19.5%, and 18.0%, respectively. The fat contents of the diets are estimated at 4%, 5%, 6%, and 7% for these four phases, respectively. Since AMEn values are given in most feed tables, the NE requirements for Ross broilers targeting live weights between 2.0 and 3.5 kg can be calculated according to the equation developed by Wu et al. (2019), Eq. (1): 2340, 2414, 2470, and 2504 kcal/kg for starter, grower, finisher 1, and finisher 2, respectively. These values represent NE/AMEn ratios of 78.66%, 79.15%, 79.68%, and 80.12%, indicating varying NE/AMEn depending on the CP and fat contents of the diets at different phases. Similarly, users can obtain the NE requirements based on AMEn values and CP levels available in the breed recommendations with their own estimated fat contents, depending on their production circumstances, such as breed and raw materials. If the equation proposed by Noblet et al. (2024) is used, the NDF content of the diet needs to be included according to the ingredients commonly used.

## 5 The benefits of an NE system

It is generally conceded that the use of an NE-based system to account for the 25%–35% feed energy lost as heat will not impose a disadvantage on feed formulation. In fact, NE offers better accuracy in predicting available energy as it accounts for the differences in efficiency of utilisation by different nutrients for maintenance and production, as opposed to the ME system, which assumes that dietary components lose energy at a similar rate during digestion and metabolism. The NE systems also pair naturally with low-protein diets, reducing the metabolic cost of deamination and N excretion. For instance, each percent reduction in dietary protein can reduce nitrogen excretion by approximately 10% (Belloir et al., 2017; Kerr and Kidd, 1999) which, in turn, leads to improved gut health due to less undigested protein reaching the hindgut (lowering necrotic enteritis risk, for instance). In addition, reducing the protein level alleviates, albeit slightly, the burden associated with heat production, especially during heat stress.

Another change that occurs when formulating on NE is the re-ranking of ingredients, where the shadow prices of fats and oils widen, favouring higher levels of their inclusion in formulation and those of vegetable proteins narrow, making them less attractive to include at higher levels. Re-ranking of ingredients is a double-edged sword. It can be costly if the producer is constrained by raw material procurement, storage or resale, having to place unnecessary nutrient or ingredient constraints to force certain ingredients into the formulation. On the other hand, as NE systems give a fairer, more realistic energy value to low-cost, alternative ingredients, the chances for such ingredients to come into formulation based on shadow prices are often improved.

A well-recognised issue with the ME system is that dietary AME values, unlike NE values, are not good predictors of bird performance, ie., FCR and growth rate. Saheb fosoul et al. (2018) demonstrated a significant correlation between dietary NE and weight gain of broilers, whereas this relationship was not seen with AME. Zou et al. (2021) used the NE system developed by Wu et al. (2019) in comparison to the AME system and found that formulating on NE values, compared with AME values, was better in predicting feed efficiency in growing chickens.

Since the NE systems give ingredient energy values that better reflect true productive value, NE-based least-cost formulation should produce diets that are more economically efficient. Published literature on the economic benefits of NE systems is scarce. But there is a substantial body of industry work on NE that is worthy of mention. In a study involving 16 broiler farms with multiple rounds, 7 million birds per round, CP Thailand compared formulations based on NE and ME and manufactured the feed in the same feed mill to avoid processing-related errors to evaluate the economic and sustainability aspects of the two energy systems in broilers. The use of NE diets resulted in savings of $2, $2, and $4 per tonne of feed in the formulation cost for the starter, grower, and finisher periods, respectively. The NE formulation allowed in more alternative ingredients, resulted in a lower feed cost per kg body weight gain, produced a lower carbon footprint (17% reduction during the finisher phase), and led the birds to retain 3%–5% more N and have a lower incidence of footpad dermatitis. The growth rate and FCR were similar between the two formulations or slightly better for the NE diets (Srichana, unpublished data).

The magnitude of improvement produced by NE in bird performance is often small but the overall benefit of an NE system for poultry production is manifold and real. Thus, despite strong interest in using an NE-based system for poultry feed formulation in some countries, its uptake in the poultry industry across the globe has been slow and low. One of the key reasons for this may be that benefits small in magnitude are difficult to detect, especially when nutritionists set wide safety margins in the nutrient specifications they use, as well as possible errors in the energy values of raw materials. It is not difficult to speculate that such issues may mask the benefits of NE, leading to hesitancy and inaction. To implement an NE system in poultry, the first step is to go back to the basics and strengthen the accuracy of the nutrient databases. The second step is then to validate the NE system of your choice using appropriately designed trials in terms of characterisation and processing of the raw materials used in the formulation(s), management and husbandry of the birds, and collection and treatment of the data. These apply to both research and industry trials. The final step is to examine the data from the entire production point of view, instead of a few performance parameters such as body weight gain and FCR.

## 6 Recommendations

Approximately 50% of GE ends up as NE. There are significant opportunities to increase this efficiency. However, to realise these opportunities, methodical steps must be taken when transitioning from the AME-based formulation to an NE-based system. Based on industry experience, five key steps are recommended.

1) Keep your data in check

Savings start with a more stringent review of the ME values of the ingredients in use. As discussed earlier, most publicly available feed tables present AMEn values, often without a clear outline of how the values were obtained, eg., the age of the animal and the assay method. Thus, it is important to cross-check the ME values in your database against the main feed tables. Of course, the ultimate cross-check is to use an appropriate bioassay (Wu et al., 2020) if conditions permit.

2) Pick the Eq.

Two principal equations are recommended, which are:

NE(kcal/kg)=0.808×AMEn(kcal/kg)–4.06×CP(%)+7.41×EE(%)(Wuetal.,2019);

NE(kcal/kg)=0.815×AME(kcal/kg)–6.21×CP+4.78×EE–5.74×NDF(Nobletetal.,2024).

The original publications (Wu et al., 2019; Noblet et al., 2024) present these equations in MJ/kg DM, whereas the current recommendations are in kcal/kg to facilitate easier calculation for those who use kcal as their energy unit, as long as the DM contents of the raw materials stay within the normal range.

3) Add NE values to your database

Once the data are deemed satisfactory and an equation is selected, it is a simple yet important step to add an NE column in the database and populate it with the converted NE values.

4) Set the requirements

The major broiler breeding companies adjust the nutrient specifications for their breeds every three to five years, according to the genetic progress occurred. The usual items of adjustment are ME contents and amino acid levels. The ME requirements are based on AMEn values. Credible NE equations are developed based on test diets that resemble practical broiler feed and take into account the factors that affect the use of AME for NE. Hence, NE requirements should be calculated using the NE equation chosen. Examples were given earlier under “NE requirements”.

5) Formulate on NE

The last step is to set the energy requirements to NE without reference to AME and formulate the diet as usual.

## 7 Conclusions

Industry experience suggests that a proper transition to an NE-based system for broiler chicken production yields several benefits, such as lower feed cost, lower food pad dermatitis, and lower emissions. In the absence of a practical and economical way to measure the NE values of individual ingredients, the use of the NE equations published by Wu et al. (2019), Tay-Zar et al. (2024), or Noblet et al. (2024), which were developed from industry-relevant NE trials and a compilation of the most recent NE measurements carried out on balanced diets and modern broilers, is recommended. An NE-based feed formulation, in general, should offer greater accuracy, better biological relevance, and improved economic efficiency compared with traditional ME systems, either AME or AMEn. Future research needs to ascertain the impact of feed additives, examine how feed components affect maintenance energy requirements, the roles of amino acids and their balance on the performance of broilers on NE formulation, and further define how TDF and its constituents influence NE in poultry. Although massive savings in production costs are not always apparent, chicken meat producers have nothing to lose by transitioning from an AME-based formulation to an NE-based formulation.

## Credit Author Statement

**Mingan Choct:** Writing – original draft, Conceptualization. **Shubiao Wu:** Writing – review & editing.

## Declaration of competing interest

Mingan Choct and Shubiao Wu are Board Member for Animal Nutrition and were not involved in the editorial review or the decision to publish this article.
